# The Long-Term Success of Mandatory Vaccination Laws After Implementing the First Vaccination Campaign in 19th Century Rural Finland

**DOI:** 10.1093/aje/kwac048

**Published:** 2022-03-15

**Authors:** Susanna Ukonaho, Virpi Lummaa, Michael Briga

**Keywords:** historical Finland, societal development, vaccination coverage, vaccination law, vaccine hesitancy

## Abstract

In high-income countries, childhood infections are on the rise, a phenomenon attributed in part to persistent hesitancy toward vaccines. To combat vaccine hesitancy, several countries recently made vaccinating children mandatory, but the effect of such vaccination laws on vaccination coverage remains debated, and the long-term consequences are unknown. Here we quantified the consequences of vaccination laws on vaccination coverage, monitoring for a period of 63 years (1837–1899) rural Finland’s first vaccination campaign against the highly lethal childhood infection smallpox. We found that annual vaccination campaigns were focused on children up to 1 year old and that their vaccination coverage was low and declined over time until the implementation of the vaccination law, which stopped the declining trend and was associated with an abrupt coverage increase, of 20%, to cover >80% of all children. Our results indicate that vaccination laws can have a long-term beneficial effect of increasing the vaccination coverage and will help public health practitioners to make informed decisions on how to act against vaccine hesitancy and optimize the impact of vaccination programs.

## Abbreviations

AICc: second-order Akaike information criterion
CI: confidence interval
CV: coefficient of variation
GAMM: generalized additive mixed model
SD: standard deviation

Infectious diseases are a major public health concern and socioeconomic burden at all ages (1), but children are by far the most vulnerable to infections (2). In many low- and middle-income countries, pertussis and measles remain among the top 10 causes of mortality for children below age 5 years (3, 4). In high-income countries, these infections are currently on the rise (5–11), despite the ready availability of vaccines.

One factor driving the increasing burden of childhood infections in high-income countries is vaccine hesitancy (5–11): partial or delayed acceptance or refusal of vaccination despite the availability of vaccines (12–14). Vaccine hesitancy is a key concern for public health institutions, because vaccination is our main tool to reduce and ultimately reach the local elimination of childhood infections (15). For the childhood infections measles and pertussis, reaching elimination requires a vaccination coverage of 95% and 98% of the population, respectively (16, 17), and such levels of vaccination coverage cannot be achieved with the current levels of hesitancy in high-income countries, especially for example, when there are spatial clusters of unvaccinated groups from which infections can re-emerge and spread to other geographic regions (18).

One strategy to combat vaccine hesitancy is to adopt vaccination laws (i.e., make vaccination compulsory). Many states in the United States and more recently in the European Union—as in Italy, France, and Germany in 2017, 2018, and 2020, respectively—have adopted vaccination laws that require children to be vaccinated against several childhood infections in order to be allowed into public schools or collective child services (19–21). These rules are enforced with fines (19, 20), but children can be exempted for medical and in some places for various nonmedical reasons, which in the United States has resulted in patchy vaccination coverage (22, 23). Hence, the extent to which vaccination laws are effective at increasing vaccination coverage is debated (24–29). So far, preliminary studies and short-term monitoring indicate that recent vaccination laws have been successful at generating a short-term increase in vaccination coverage in Italy and France (19, 21) and in California, where a recent vaccination policy eliminated nonmedical exemptions from school entry requirements (30). However, the extent to which vaccination laws increase vaccine uptake in different countries and sociodemographic contexts remains unknown.

In this study, we quantified the vaccination coverage, the long-term impact of a mandatory vaccination law on the vaccination coverage, and its spatiotemporal dynamics during Finland’s first vaccination program against smallpox in the 19th century. In brief, smallpox was one of the most lethal diseases in human history, affecting especially European and Native American populations (31–33). For example, in our study population in 18th and 19th century Finland, smallpox was a major cause of death in children, killing up to 15% of Finland’s population around 1800, before the introduction of vaccines (34–36).

**Figure 1:**
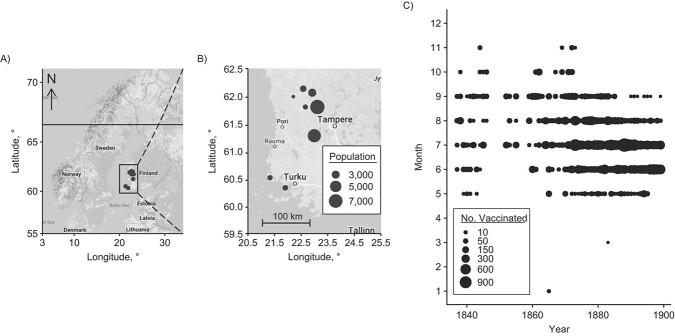
Geographical and temporal distributions of the data, showing a map of the 8 rural parishes in southwest Finland, with a map of Northern Europe on the left (A) and a zoomed-in map of southwest Finland on the right (B), and the predominant seasonal timing of annual vaccination campaigns (1837–1899) occurring in summer (C).

Little is known about how the smallpox vaccination campaign was implemented in the 19th century and to what extent it was successful (32), especially in rural areas where vaccine uptake remains low even today in many parts of the world (37). For the purpose of this study, we digitized a series of unique historical vaccination records (63 years, 1,800 pages, and over 46,000 individuals) maintained by local churches and district doctors from the first vaccination campaign against smallpox in 8 rural parishes of Finland (Figure 1A–B, Web Figure 1, available at https://doi.org/10.1093/aje/kwac048). This study has 2 aims. First, we describe the seasonal and annual dynamics of the smallpox vaccination campaigns in rural Finland and which age groups were targeted. Knowing the age at vaccination is relevant for vaccine effectiveness; late vaccination diminishes protection against infection, thereby enabling the transmission of infection, while early vaccination can result in ineffective vaccination due to interference with maternal antibodies (38) in addition to considerable negative side effects (39, 40). Second, we tested 2 hypotheses, namely: Did the vaccination law cause: 1) a long-term increase in mean vaccination coverage and 2) a long-term decrease in variance in vaccination coverage? Increasing vaccination coverage and stabilizing its spatiotemporal variance are important because high variance can create pockets of susceptible persons from which epidemics can recur (41). As vaccination behaviors tend to cluster within populations, highly vaccine-hesitant communities may not reach herd immunity, thus posing a risk of disease outbreak (18). The high vaccine hesitancy in the 19th century together with the rare long-term monitoring of vaccination coverage after the vaccination law provide a unique opportunity to quantify the long-term impact of vaccination laws in a high-opposition setting, which is not yet possible in countries that recently implemented vaccination laws.

## METHODS

### Study population and data collection

Finland’s first vaccination campaign was met with high hesitancy from the public (42, 43). It started in 1802, soon after the development of world’s first vaccine, by the British physician Edward Jenner in 1796 (44). Historical records describe that, during our observation period, the distribution of vaccines improved gradually (42, 43). In 1825 the country was divided into vaccination districts, which kept records of vaccinated and nonvaccinated citizens. Each district was supervised by a district doctor, who received annual reports from the vaccinators, who were physicians, clergymen, and public officers. As the opposition against vaccines remained strong despite efforts to reassure the public, a mandatory-vaccination law was adopted in 1883, which required parents to vaccinate children under the age of 1 year, and it was gradually enforced in 1885–1890 and onward with a fine (42).

We photographed and digitized vaccination records manually from extensive church records held in Finnish national and provincial archives. These data include details of the villagers and their lives, required by law to be collected by local clergymen from 1749 onward. The clergymen recorded the name, address, parents’ occupation, age, and the level of success of the vaccination (as indicated by reported reaction to the vaccine: effective, mild, or no reaction) for each person receiving the vaccination at a given time, as well as previously vaccinated, absent, or refused individuals (Web Figure 1). Based on the accessibility and completeness of historical records, we obtained data from 8 rural parishes (Honkajoki, Ikaalinen, Jämijärvi, Karvia, Kustavi, Parkano, Rymättylä, and Tyrvää) from 1837–1899, but we lacked vaccination records from 1847–1851, 1854, and 1856–1858 for all parishes excluding Ikaalinen and Tyrvää and from Kustavi before 1862 and Rymättylä before 1865 (Web Figures 2 and 3). These records include information on a total of 46,232 vaccinations, 26,328 and 19,935 records from the before-mandatory and mandatory eras, respectively (Table 1).

**Table 1:** Overview of the Vaccination Data for All 8 Parishes for Both Eras, Before Mandatory Vaccination and With Mandatory Vaccination, Southwest Finland, 1837–1899

**Era**	**Years**	**Parish Size**	**No. Vaccinated**	**Average No. Vaccinated Annually Per Parish**	**AverageVaccinationMonth**		**Average % Vaccinated Ages <1 Year**	**Average % Vaccinated, Ages 1–4 Years**	**Interannual and Inter-Parish SD**	**Interannual and Inter-Parish CV**
									**Month**	**95% CI**
Before mandatory vaccination	1837–1882	2,600	26,328	108	7.40^a^	7.38, 7.42	68.1	16.3	27.8	40.9
Mandatory-vaccination era	1883–1899	3,700	19,935	148	6.72^b^	6.71, 6.73	87.5	10.3	21.5	24.6

Abbreviations: CI, confidence interval; CV, coefficient of variation; SD, standard deviation.

^a^ Mid-July.

^b^ Late June.

In order to estimate the number of vaccinated children per age group, we gathered birth and death records for each parish from the national and provincial archives, the Genealogical Society of Finland, and Finland’s Family History Association digitized archives. These records consisted of yearly births and deaths per age cohort (e.g., <1, 1–2, 3–4 years), which we used to calculate the number of individuals per age cohort per parish. We estimated the vaccination coverage based on the church records and total cohort sizes by calculating the yearly proportion of vaccinated per cohort and parish from 1837–1899. There were a few years with >100% vaccination coverage, which was likely the result of population movement. Birth records are unlikely to be the cause of vaccine coverage reaching over 100% as these have been validated by independent studies (45, 46). For every 5–10 years we had review tables of age-specific cohort sizes per parish from the historical records, which confirmed our annually estimated cohort sizes.

### Statistical analyses

#### Seasonality and age distribution of the vaccination campaigns.

The first aim of this study was to describe the occurrence and seasonal dynamics of the vaccination campaigns. To detect whether vaccination campaigns were seasonally recurring, we conducted wavelet analysis. In brief, wavelet analysis decomposes time-series data using functions (wavelets) simultaneously as a function of both time (year of observation) and period, with a period of 12 months indicating a yearly seasonally recurring pattern (47). We performed all statistical analyses using R, version 3.6.1 (R Foundation for Statistical Computing, Vienna, Austria) (48). For the wavelet analyses, we fitted a Morlet wavelet with the function “analyze.wavelet” of the package “WaveletComp” (49). To minimize the impact of any influential data points, we smoothed and detrended the data following standard protocol (49). We determined statistical significance by comparing the observed periodicity from the data with that of 1,000 “white noise” simulated data sets with (95%) significance level.

We then describe the age structure of the vaccination campaigns. For these analyses, we used a matrix with the number of vaccinated per age group per year and divided age into 4 groups (in years: <1, 1–2, 3–4, ≥5). We tested for the preference toward children under 1 year old using nonparametric permutation tests with the function “independence_test” of the package “coin” (50) based on 10,000 permutations. We also describe the age at first vaccination for 12,589 individuals with exact birth dates.

#### Spatiotemporal dynamics of vaccination coverage.

To quantify the dynamics in vaccination coverage over time and across parishes and whether the vaccination law affected these dynamics, we used generalized linear mixed models (GLMs) and generalized additive mixed models (GAMMs). We fitted 4 statistical models with, as the dependent variable, either: 1) the number of vaccinations, 2) the vaccination coverage, 3) the parish-level annual standard deviation (SD) in vaccination coverage, or 4) the parish-level annual coefficient of variation (CV) in vaccination coverage (for details, see Web Table 1). In all of these models, the predictor variable was year standardized to the mean of zero and an SD of 1.

We fitted GLMs with the functions “glm,” “lme,” and “gls” of the packages “stats” (32) and “nlme” (51). These gave consistent results and we here present the results using “glm” for the number of vaccinated (Poisson-distributed counts; model A), “lme” for vaccination coverage (Gaussian-distributed; model B) and “gls” for parish-level SD and CV (Gaussian-distributed with temporal autocorrelation, see below, models C and D). To quantify the statistical “significance” of predictor variables, we compared the model fits on the data with the second-order Akaike information criterion (AICc; (52, 53)), using the function “AICc” of the package “MuMIn” (54). In brief, model selection ranks the models based on their AICc value, where better-fitting models are indicated by lower AICc, models within 4 ΔAICc are considered plausible, and increasingly implausible up to 14 ΔAICc after which they are implausible (52, 53).

To account for the fact that people are clustered into parishes, we included parish identity as a random intercept in model B, the vaccination coverage analyses, which improved the model fit (ΔAICc = −29 in Web Table 1B). To have an idea of the importance of parish-level differences, we reported the proportion of variance in the model explained by the random intercept and estimated the repeatability of parish-level differences with the function “rpt” of the package “rptR” (55). The repeatability is an intraclass correlation coefficient that captures the between-parish variance (by parish identity as a random intercept) relative to the total variance and ranges from zero (no parish-level differences) to 1 (all variance in the dependent variable is explained by parish-level differences). We estimated the 95% confidence intervals (CIs) around the repeatability based on 1,000 bootstraps.

All model residuals were correctly distributed and fulfilled all other assumptions as checked with the function “simulateResiduals” of the package “DHARMa” (56). In model B, there was, however, heteroscedasticity as identified with the functions 1) “testQuantiles” from the package “DHARMa” (56), and 2) the Breusch-Pagan test performing a linear model using as dependent variable the squared value of the conditional residuals of the model B and standardized year as predictor variable (57). In model B, we corrected for heteroscedasticity by including the variance power (“varPower”) in the function “weights” (58, 59). We then tried to identify 2 sources of changes in heteroscedasticity by decomposing vaccination coverage into a parish-level component and an annual component. For the parish-level component, we used as the dependent variable the annual parish-level SD and CV in vaccination coverage (respectively, models C and D, Web Table panels 1C and 1D). To identify whether there was an annual component in heteroscedasticity, we used the Breusch-Pagan test as described above on the annual mean in vaccination coverage (excluding parish-level variance) and using before-mandatory and mandatory era as a 2-level categorical predictor.

We controlled for temporal autocorrelation following Woods (60) by including standardized year as an autoregressive factor of order 1 (corAR1). In models A, number of vaccinated, and B, vaccination coverage, this worsened the model fit (ϕ = 0.57 ± 0.25, ΔAICc = +155 for model B in Web Table 1B), indicating no temporal autocorrelation, a conclusion also supported with the function “acf” (ACF < 0.1) of the package “stats” (32). For these models, we have hence presented the results without temporal autocorrelation, but analyses including temporal autocorrelation gave consistent conclusions (results not shown). Models C and D, respectively, parish-level SD and CV in vaccination coverage, were autocorrelated and thus we included the corAR1 autocorrelation structure in these models.

To investigate whether there were abrupt changes in vaccination coverage or its spatiotemporal variance that coincided with the start of the vaccination law, we fitted threshold models following Douhard et al. (61) and Briga et al. (62). In brief, for each model (A–D), we tested a series of threshold models, with 1-year intervals between each threshold, and identified the best-fitting threshold model based on the model’s AICc value. To identify whether threshold models were appropriate at all, we compared the model fit of the best-fitting threshold model with that of a linear model without threshold (Web Table 1A–1D). We identified the confidence interval around the threshold year by including all models within 4 ΔAICc of the best-fitting threshold model (61, 62), abbreviated as 4AICCI (Web Figure 4A–D). To confirm the results of threshold models, we performed all analyses also using GAMMs, with the function “gamm” of the package “mgcv,” and to identify the year of change (inflection points) we used their derivatives with the function “fderiv” of the package “gratia” (63). All GAMM analyses confirmed the conclusions obtained from the threshold models (Web Figure 5A–5H).

## RESULTS

### Seasonality and age distribution of the vaccination campaign

Here we present data covering 8 rural parishes in southwest Finland (Figure 1A–B), with 26,328 records (57%) during 46 years of the era before mandatory vaccination, from 1837–1882 (average 572 records per year), and 19,935 records (43%) during 17 years of the mandatory-vaccination era, from 1883–1899 (average 1,173 records per year; Table 1).

During both eras, vaccination campaigns started in May or June, occurred predominantly in summer, and often ended in August or September (Figure 1C). This seasonal pattern was confirmed by wavelet analysis, where we observed a statistically significant annual vaccination periodicity (*P* < 0.05), both on average over the whole observation period (*P* < 0.05, Web Figure 2A) and in the time dependent analyses (*P* < 0.05, Web Figure 2B).

**Figure 2:**
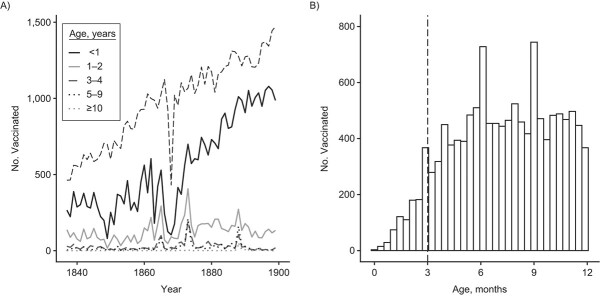
The number vaccinated per age group and age distribution for children under the age of 1 year, southwest Finland, 1837–1899. Smallpox vaccinations had a preference toward the youngest age groups, with no apparent minimum age. A) The dashed black line indicates the number of annual births in the 8 study parishes. B) The dashed vertical line indicates contemporary recommendation for age at first vaccination, which is 3 months in Finland for most vaccines. For smallpox, the recommended age at first vaccination is 1 year (39).

We then investigated the age distribution of the people vaccinated during the campaigns. There was a clear preference toward children under age 1 year, with on average 69% of the records in the before-mandatory-vaccination era being for children under age 1, 21% aged 1–2 years, 5.3% aged 3–4 years, and 4.5% aged 5 years old or older (χ^2^ = 1,143, *P* < 0.0001, Figure 2A). In the mandatory-vaccination era, the preference toward 1-year-olds was 10% higher at 80% of the records, and this increase was statistically significant (χ^2^ = 39.73, *P* = 2.9 × 10^−10^, Figure 2A). We did not observe a lower limit for the age at first vaccination (Figure 2B): 872 of the 34,496 records (2.5%) of the children under the age of 1 were below the age of 3 months. Thus, there was a preference to vaccinate before age 1, which increased with time and with no apparent lower age limit.

### Spatiotemporal dynamics of vaccination coverage

The number of vaccinations increased with time (Figure2A, Web Table 1A). Threshold models showed that this increase accelerated after the break point in 1871 (1871< 4AICCI < 1871; Web Figure 4A), and this result was supported by GAMMs (Web Figure 5A and 5B). This break point coincided with the time shortly after the drop in births during the famine years of 1867–1868. Because vaccination dynamics can be driven mostly by the dynamics of births (Figure 2A), we studied birth-independent spatiotemporal dynamics of vaccinations by using vaccination coverage, that is, the number of individuals vaccinated relative to the number of individuals in the age category, and focused the analyses below on children up to age 1 year.

**Figure 3:**
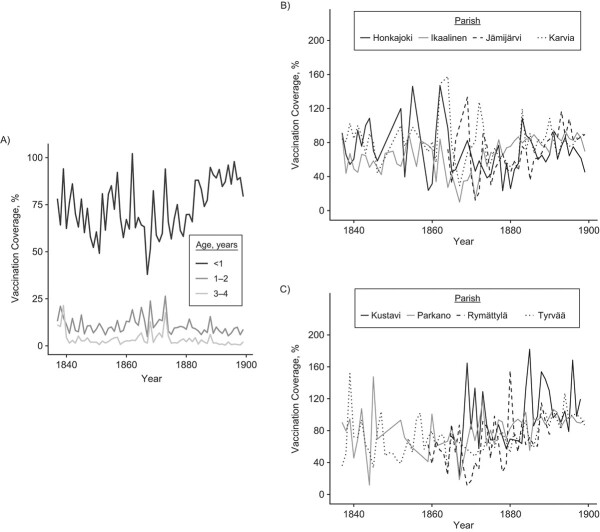
Vaccination coverage increased after the introduction of the vaccination law in 1883 (A) together with the decrease in the variance between parishes, which, for illustrative purposes, is shown in 2 plots, each showing 4 of the 8 parishes (B–C), southwest Finland, 1837–1899.

**Figure 4:**
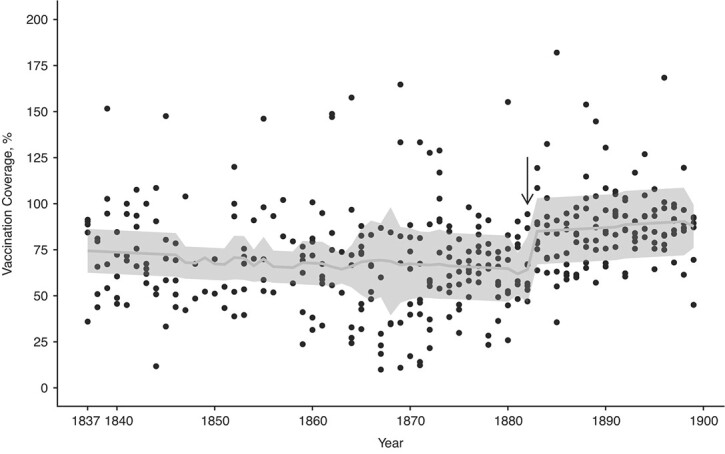
The temporal dynamics in vaccination coverage of children up to age 1 year (southwest Finland, 1837–1899) are best captured by a threshold model with an increase in vaccination coverage after 1882. Before the threshold, indicated by the black arrow, the vaccination coverage decreases with time, whereas after the threshold there is an increase. However, the 95% confidence interval after the threshold overlaps with zero, and hence the increase is not statistically significant. The predicted values are plotted as a light gray line against the black observed data points, and the gray bands represent the 95% confidence intervals.

We tested the hypothesis that the vaccination law increased vaccination coverage. Before mandatory vaccination, vaccination coverage was on average 68% (SD, 27.8; 18.7 < (95% CI) < 147) and in the mandatory-vaccination era this increased by 20% to 88% (SD, 21.5; 57.4 < (95% CI) < 143; Figure 3). Analyzing these time series with threshold models showed an abrupt increase in vaccination coverage in 1882 (1882 < 4AICCI < 1882; ΔAICc relative to a linear increase = −30.4; Web Table 1B; Figure 4, Web Figure 4B). Before the threshold the vaccination coverage decreased with time, while after the threshold we observed an increase. Before the threshold, the 95% CI of the coefficient does not overlap with zero and hence is statistically significant, but after the threshold the 95% CI overlaps with zero and hence is not significant (respectively: β_1_ = −4.27, −8.35 < (95% CI) < −0.20, *P* = 0.04; β_2_ = 9.22, −6.89 < (95% CI) < 25.33; Web Table 1B). GAMMs supported the conclusions of threshold models with the gamm derivative indicating the steepest increase in coverage in 1882 (Web Figure 5C–D). Hence, the implementation of the vaccination law was associated with: 1) a stop in declining coverage and 2) an abrupt 20% increase in coverage, which was 3) persistent in the long-term.

We then tested whether the variance in vaccination coverage changed over time. A Breusch-Pagan test indicated a decrease in variance (ΔAICc = −15.49). We tried to decompose whether this decrease in variance was due to a spatial (between parishes) and/or temporal (between years) component. Vaccination coverage varied substantially and consistently between parishes; parish identity captured 9.0% of the variance in vaccination coverage during the era before mandatory vaccination, which increased to 46% in the mandatory-vaccination era (model in Web Table 1B) with a repeatability of, respectively, 0.083 (0.00 < (95% CI) < 0.21) and 0.32 (0.069 < (95% CI) < 0.55). The parish-level SD and CV in vaccination coverage decreased over time and showed an abrupt change around 1873 (SD, 1869 < 4AICCI < 1876; CV, 1872 < 4AICCI< 1874; Web Table 1C–D; Figure 3B, Web Figure 4C–D), and GAMMs confirmed these conclusions (Web Figure 5E–H). The interannual SD decreased as well with time, from 12.94 in the era before mandatory vaccination to 6.48 in the mandatory-vaccination era (Breusch-Pagan test, *P* = 0.46). Hence the variance in vaccination coverage stabilized between parishes and over time, but the parish-level stabilization started approximately 10 years before the introduction of the vaccination law.

## DISCUSSION

Common, preventable childhood infections are on the rise in many contemporary high-income countries following decreases in vaccination coverage, which has led to laws mandating vaccination against infections such as measles and pertussis in, for example, Europe, many US states, and Australia. However, we know almost nothing about the long-term effects of vaccination laws on vaccination coverage and its dynamics. In this study, we show that Finland’s first vaccination campaigns in rural parishes were executed mainly during the summer, often in the middle of farming season, and most of the vaccinated individuals were under the age of 1 year. We found an abrupt increase in vaccination coverage that coincided with the start of a mandatory-vaccination law and persisted for at least 15 years. However, we also found differences in vaccination coverage between parishes, and although spatiotemporal variance in vaccination coverage decreased with time, this variance persisted, and its sudden decrease 10 years before the vaccination law appeared not to be associated with the vaccination law. Here we discuss 3 implications of our results for the impact of vaccination campaigns and vaccination policy.

First, low vaccination coverage in rural areas is a challenge that contemporary vaccination campaigns try to solve, especially in low- and middle-income countries (64–66). Increasing vaccination coverage in these areas is important because spatial heterogeneity in vaccination coverage creates low-vaccination clusters, which can become a source of outbreaks that spread to other areas (18). Our finding of repeatable parish-specific vaccination coverage show that vaccination coverage is a geographically local characteristic. The reasons behind the poor and fluctuating coverage in rural communities are complex (12, 64, 65), but at least 2 possibilities stand out. First, remote parishes are often more difficult to access and possibly less well managed than more urban parishes (67). Second, people living in remote rural parishes might be less motivated or more hesitant to vaccinate (68, 69). We observed a decline in the spatiotemporal variation in vaccination coverage, indicating improvements in the management of the vaccination campaign and reduction in disparity between the parishes. Although vaccination law probably contributed to this decline, it started a decade earlier and shortly after the famine years (1867–1868), which suggests that it is not directly associated with the vaccination law itself and is more likely due, for example, to improved accessibility in certain rural areas.

In addition, one notable characteristic of the campaign was that children seemed to be vaccinated without a lower age limit. Smallpox epidemics were still prevalent during the 19th century, and as vaccinators traveled to rural areas only once a year, early vaccination may have been the optimal solution at the time. However, we now know that early vaccination can be ineffective, for example, through maternal blunting, where maternal antibodies of vaccinated mothers interfere with infant immune response and lower the infant’s responsiveness to vaccines (38). Hence, for example, in contemporary societies a recommended age at first vaccination is at least 3 months for several childhood infections and 1 year for smallpox (39).

Finally, mandatory vaccinations have been one of the strategies considered to combat low vaccination coverage, but this approach has always been controversial and associated with opposition (70). Our results show an abrupt increase in vaccination coverage at the introduction of the vaccination law. Similarly, short-term studies in contemporary societies observed immediate increases in vaccination coverage after the introduction of vaccination laws (19, 71), and our study suggests that the observed increases will persist on a longer term. Moreover, there were no major lethal smallpox outbreaks after 1882 (Web Figure 6), reflecting that vaccination together with, for example, general improvements in living conditions (72) diminished the lethal burden of smallpox.

Our study has several limitations. First, instead of vaccine hesitancy, vaccine delivery can be a major factor affecting vaccination coverage (73). In our study population, vaccine uptake likely improved at least in part due to better distribution and management of the vaccination campaign. We cannot exclude sudden organizational changes of vaccination campaigns, but historical records indicate that organization improved gradually during our observation period (42, 43). This is also indicated by the changes in CV and SD in vaccination coverage, which occurred 10 years prior to the mandatory-vaccination law, which probably reflects an improvement in Finland’s access to vaccines. Thus, in our study, the vaccination law and associated fines constitute just one factor that increased vaccination coverage. Second, our study population was limited to 8 rural towns from western Finland. As vaccination campaigns may differ between cities or rural parishes, it is unclear to what extent we can extrapolate the characteristics of the vaccination campaign and the response to the vaccination law in time and space. Third, as parishes did not decline anybody wanting to get vaccinated, our estimates for the number of vaccinated also include temporary travelers from other parishes, which results in some data points showing >100% vaccination coverage, indicating an overestimation of the vaccination coverage of the locals. Thus, vaccination campaigns were probably not quite as successful before or after the vaccination law as our estimates indicate.

The reasons behind changes in vaccine uptake remain complex, and hence measures other than vaccination laws—such as training medical professionals about vaccines, actively recommending vaccines, and good communication with the lay public—are also needed to increase vaccination coverage (29, 74). Implementing vaccination laws with clear and consistent communication can help reach infection-specific thresholds for herd immunity and subsequent elimination (41, 75). Our study indicates that vaccination laws will be an effective long-term tool in the public health battle to increase vaccination coverage and to work toward the elimination of childhood infections, even in sociodemographic contexts (rural areas, periods before introduction of good public health care) where vaccines are often met with higher reluctance than in urban areas.

## ACKNOWLEDGMENTS

Author affiliations: Department of Biology, University of Turku, Turku, Finland (Susanna Ukonaho, Virpi Lummaa, Michael Briga); and Infectious Disease Epidemiology Group, Max Planck Institute for Infection Biology, Berlin, Germany (Michael Briga).

V.L. and M.B. contributed equally as last authors.

This work was funded by the University of Turku Graduate School, the Ella & Georg Ehrnrooth Foundation, the Academy of Finland (292368), the European Research Council (CoG 648766), and NordForsk (104910).

Data availability: The data set is available from the corresponding author.

Many thanks to Kimmo Pokkinen, Sara Itkonen, and Tuija Koivisto for help with historical data; to the National Archives of Finland for allowing us to digitize and use the vaccination records; and to Finland’s Family History Association and the Genealogical Society of Finland for access to the birth and death data. We are grateful to the Nordemics Consortium for useful discussions.

Conflict of interest: none declared.

## Supplementary Material

Web_Material_kwac048
