# Supplementary material for: The Long-Term Success of Mandatory Vaccination Laws After Implementing the First Vaccination Campaign in 19th Century Rural Finland
Source: Am J Epidemiol. 2022 Mar 15;191(7):1180–9. doi: 10.1093/aje/kwac048 (PMC9440364; doi:10.1093/aje/kwac048)
Supplement: Web_Material_kwac048 [file web_material_kwac048.pdf]

Web Material

The long-term success of mandatory vaccination laws at implementing the first vaccination campaign in 19th century rural Finland

Susanna Ukonaho, Virpi Lummaa, and Michael Briga

Table of contents:

Web Figure 1 . . . . .pg. 2

Web Figure 2 . . . . .pg. 2–3

Web Figure 3 . . . . .pg. 3

Web Table 1 . . . . .pg. 4

Web Figure 4 . . . . .pg. 5

Web Figure 5 . . . . .pg. 6

Web Figure 6 . . . . .pg. 7

Reference. . . . . .pg. 7

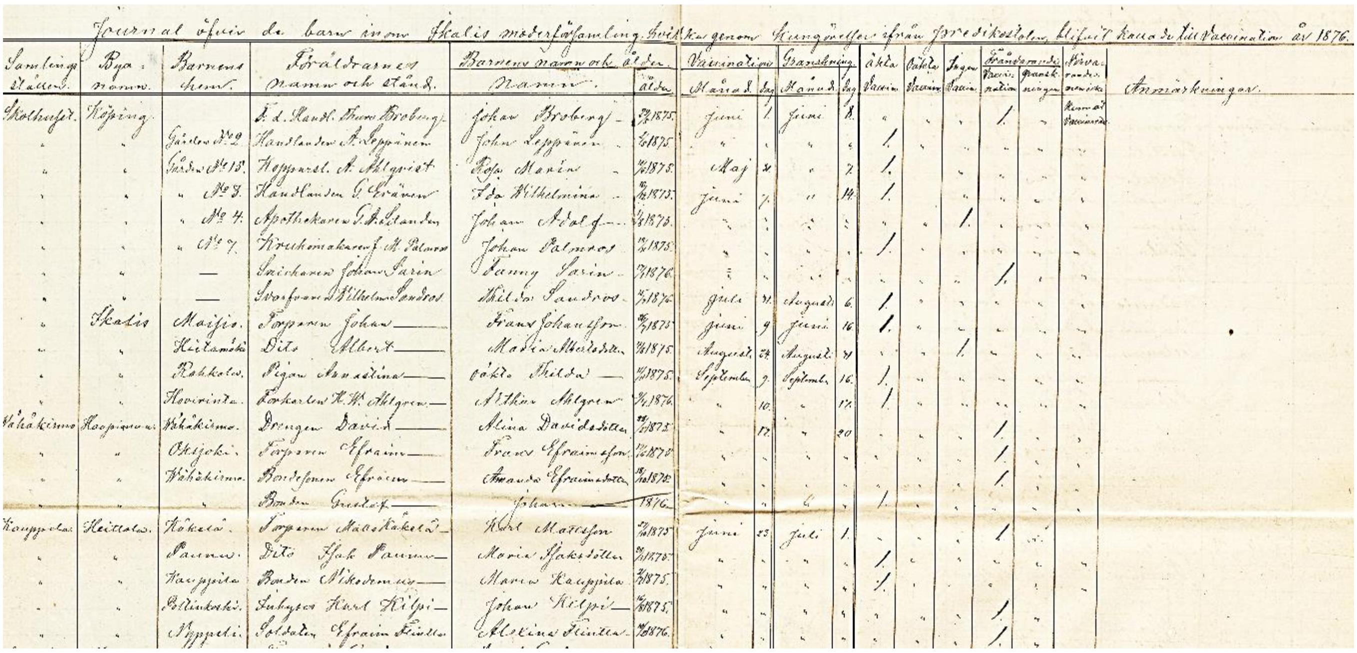

Web Figure 1. Illustration of Finnish historical vaccination records (Community: Ikaalinen, year: 1876, page 1). Columns represent (from left to right): I, ii, iii: Location, village and house; iv: Name and occupation of the parent (mother or father); v, vi: Names and birthdate of person called for vaccination; vii, viii: date of vaccination; ix, x: date of follow-up examination; xi-xiii: success of vaccination (successful, partial or no reaction); xiv-xv: absent from vaccination or follow-up examination. People who disagreed to vaccination are symbolised by ‘/’ or ‘x’ and do not have a date (not on this page); xvi: remarks and notes.

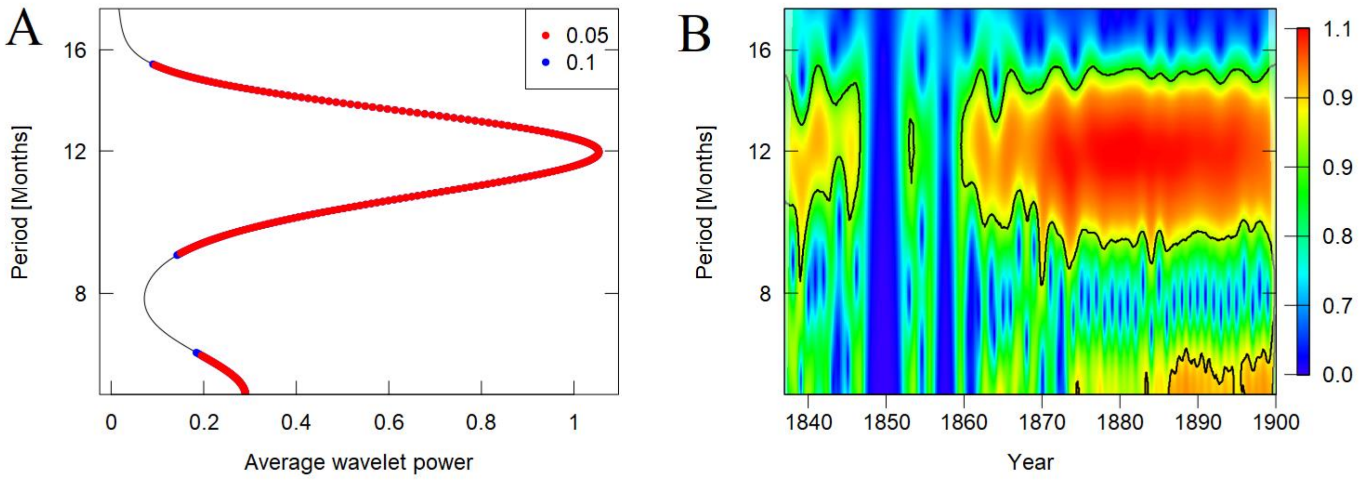

Web Figure 2. Wavelet periodogram (A) and wavelet power spectrum (B) for season at vaccination show a consistent average around one year, southwest Finland, 1837–1899. A) A smaller peak can be observed at 6 months. Red dots represent significance at the 5% level and blue dots 10% level. The axis describe period in months on the y-axis and wavelet power levels on the x-axis. B) Legend describes wavelet power levels, where red indicates strong periodicity and

green to blue signals weak periodicity. The blue gaps around 1850-1860 represent a gap in the data from all parishes at that time. The black line highlights the one-year mark in both plots.

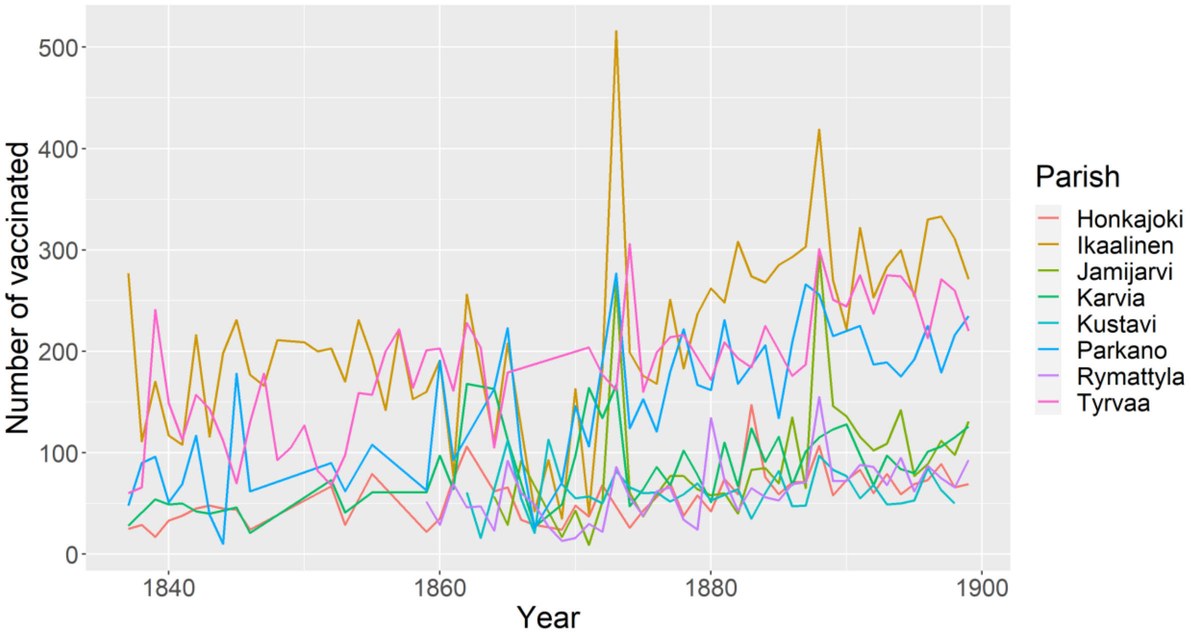

Web Figure 3. The number of vaccinated individuals increased towards the end of the 19th century, southwest Finland, 1837–1899.

Web Table 1. The model selection table indicated a quadratic increase in the number of vaccinated (count), vaccination coverage and coefficient of variation over time, but was not a significant predictor in standard deviation models. The table describes model type, model intercept, threshold intercept (Intercept2), scaled year coefficient, scaled threshold year coefficient (year2), period (before vs. after the law), model AICc, delta AICc, Akaike weight and threshold year for each dependent variable. The vaccination coverage model in B includes parish as a random variable. Best fitting models are indicated in italic.

	**Model output**				**Model selection**			
**Model**	**Intercept**	**Intercept2**	**year**	**year2**	**AICc**	**ΔAICc**	**weight**	**threshold**
A) Vaccination count								
Linear	6.51		0.43		4892.08	995.02	0.00	
No year-related change	6.60				12781.81	8884.75	0.00	
Threshold	*6.22*	*0.60*	*0.14*	*0.041*	*3897.06*	*0.00*	*1.00*	*1871*
B) Vaccination coverage								
Linear	63.33		8.21		3525.57	48.84	0.00	
No year-related change	62.82				3552.65	75.91	0.00	
Threshold	*54.13*	*21.21*	*-3.02*	*10.45*	*3476.73*	*0.00*	*1.00*	*1882*
C) Parish-level standard deviation in vaccination coverage								
Linear	23.29		-1.70		475.92	12.22	0.00	
No year-related change	23.26				477.60	13.90	0.00	
Threshold	*29.98*	*-8.73*	*5.56*	*-7.05*	*463.70*	*0.00*	*1.00*	*1873*
D) Parish-level coefficient of variation in vaccination coverage								
Linear	32.49		-3.74		520.38	30.13	0.00	
No year-related change	32.43				524.63	25.88	0.00	
Threshold	*48.09*	*-15.70*	*13.57*	*-21.17*	*494.50*	*0.00*	*1.00*	*1873*

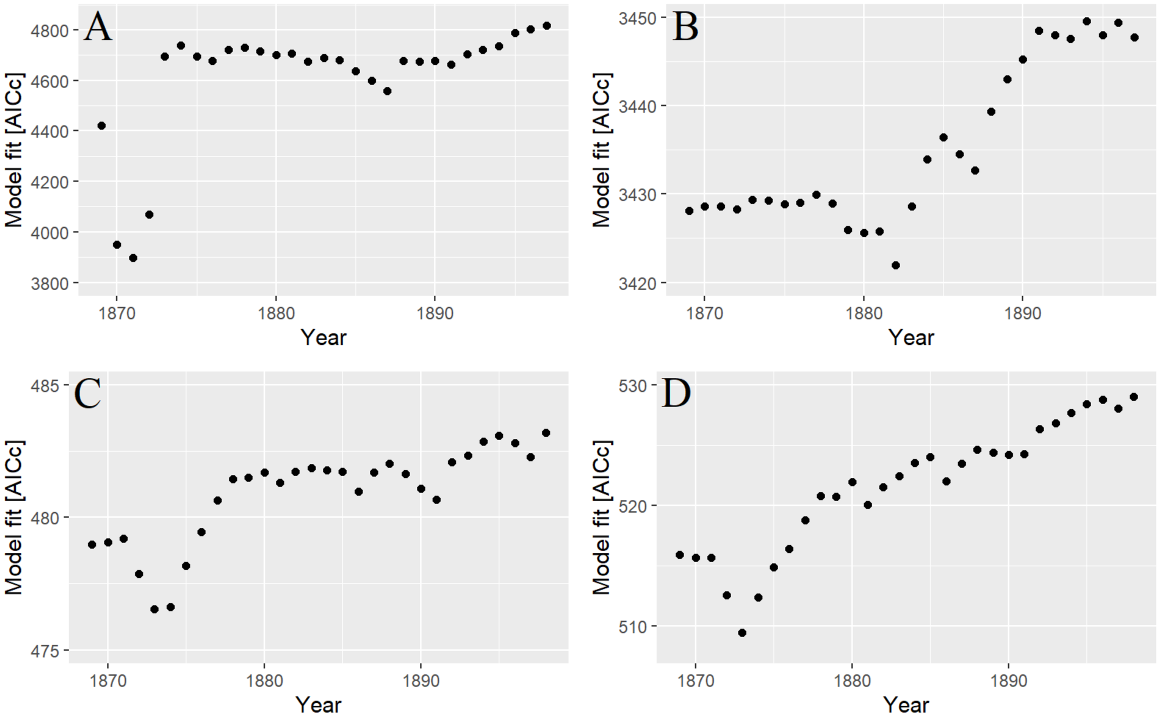

Web Figure 4. The number of vaccinated showed statistically significant threshold in 1871 (A, Web Table 1A), while the vaccination coverage showed a statistically significant threshold in 1882 (B, Web Table 1B). Both parish-level SD and CV models showed a statistically significant threshold in 1873 (C&D, Web Table 1C&D).

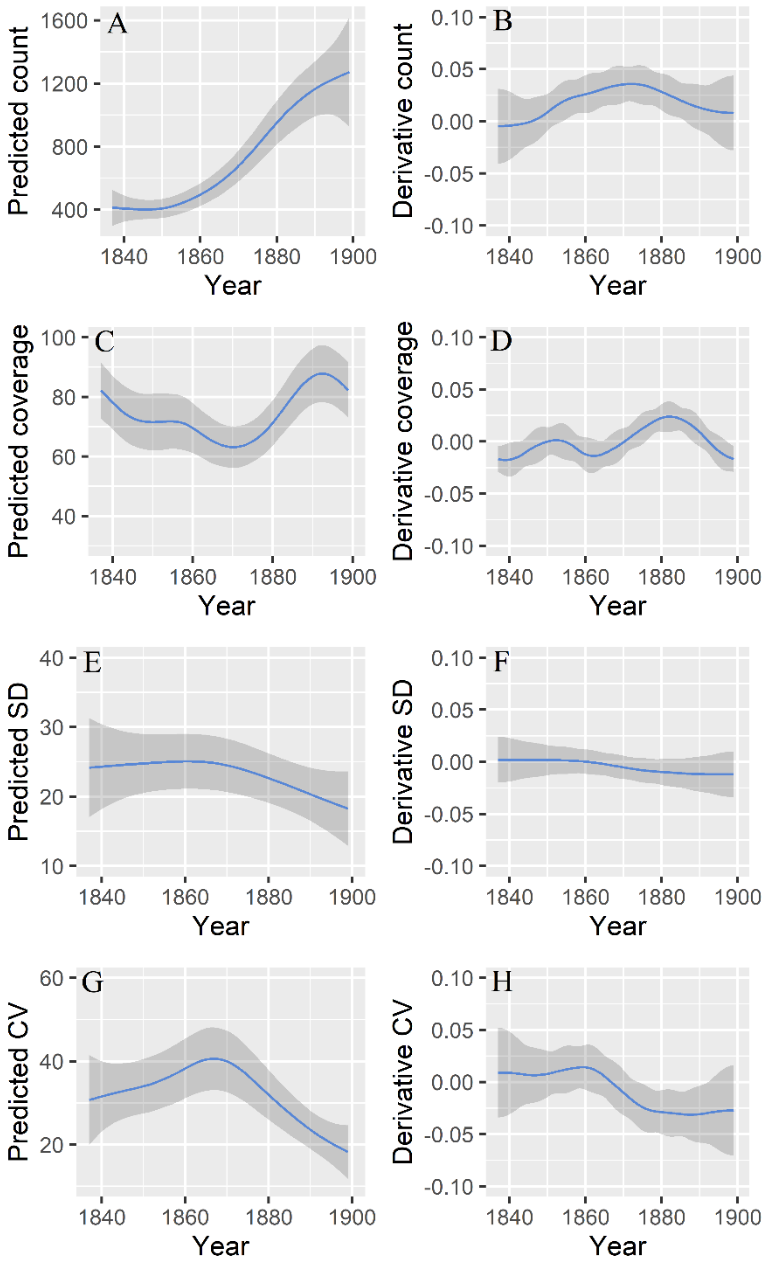

Web Figure 5. Gamm models show the steepest increase for number of vaccinated in 1871 (A) and vaccination coverage in 1882 (C), and for both parish-specific SD and CV a decrease starting from 1873 onwards (E&G), southwest Finland, 1837–1899. Shown here are the predicted values (A, C, E, G) and the derivatives of the GAMM models (B, D, F, H) for vaccination count (eq. model Web Table 1A; A&B), vaccination coverage (eq. model Web Table 1B; C&D) and parish-specific SD (eq. model Web Table 1C; E&F) and parish-specific CV (eq. model Web Table 1D; G&H). Vaccination coverage models included parish identity as a random factor. All models used autocorrelation structure AR1 for year and estimated 95% confidence intervals (grey band) around the predicted slopes (blue line).

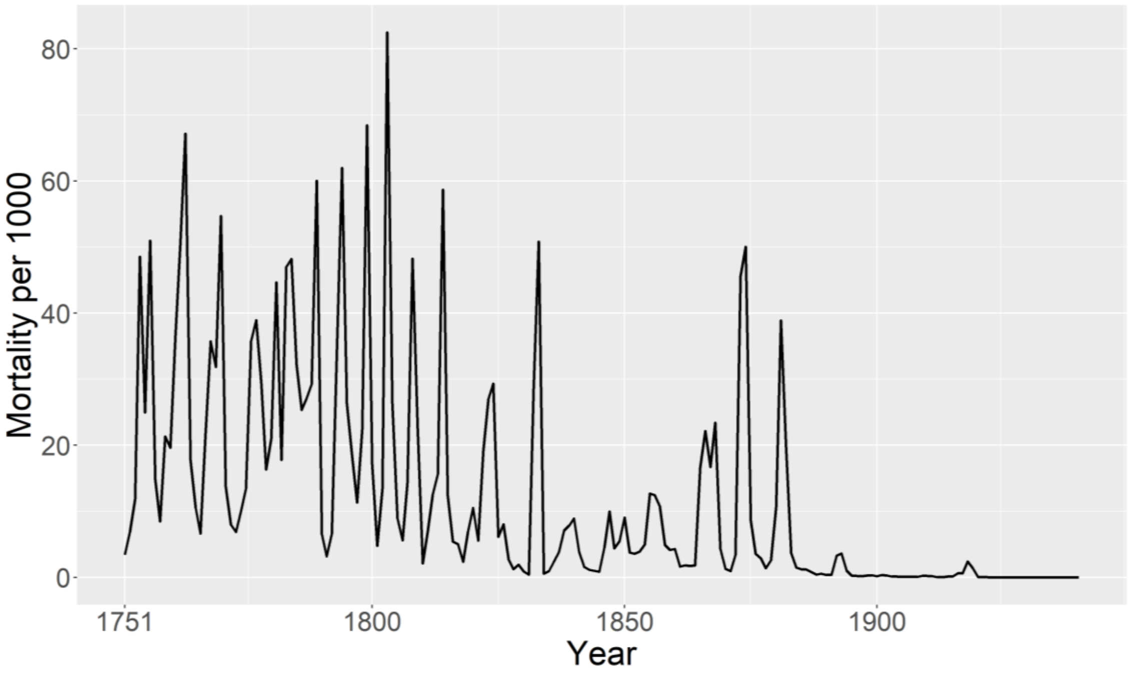

Web Figure 6. Smallpox mortality in Finland 1751–1940. Original data from Pitkänen, Mielke & Jorde 1989 (Appendix Table 1) (1).

Reference

1. Pitkänen KJ, Mielke JH, Jorde LB. Smallpox and its eradication in Finland: implications for disease control. Popul Stud (Camb). 1989;43(1):95-111. doi:10.1080/0032472031000143866
